# Comparison between point‐of‐care testing from capillary samples and conventional laboratory testing from venous samples for white blood cells and C‐reactive protein in a pediatric outpatient setting

**DOI:** 10.1002/jgf2.741

**Published:** 2024-12-15

**Authors:** Yasutaka Kuniyoshi, Takeru Kimoto, Haruka Tokutake, Natsuki Takahashi, Azusa Kamura, Makoto Tashiro

**Affiliations:** ^1^ Department of Pediatrics Tsugaruhoken Medical COOP Kensei Hospital Hirosaki Aomori Japan; ^2^ Department of Social Services and Healthcare Management International University of Health and Welfare Otawara Tochigi Japan

**Keywords:** capillary blood, child, C‐reactive protein, point‐of‐care, white blood cell

## Abstract

**Background:**

Studies on the accuracy of point‐of‐care (POC) testing using capillary samples are scarce. Therefore, this study aimed to assess the analytical accuracy of POC testing for white blood cell (WBC) and C‐reactive protein (CRP) using capillary samples compared with conventional central laboratory testing using venous samples in a pediatric ambulatory care setting.

**Methods:**

This was a retrospective study including patients younger than 18 years who underwent concurrent WBC and CRP evaluations via capillary and subsequent venous sampling within a 2‐h window. Capillary and venous blood samples were collected using finger prick and standard venipuncture techniques, respectively. Capillary blood analysis was performed using a Microsemi CRP device. Venous samples were measured in the hospital's central laboratory. The agreement between the capillary POC and venous laboratory results was evaluated using Bland‐Altman analysis.

**Results:**

A total of 277 pediatric patients were included in this study. The median age of the participants was 1 year (interquartile range: 0–2 years). The mean difference between the capillary and venous measurements for WBC was −18 × 100/μL with 95% limits of agreement of −73 × 100/μL to 37 × 100/μL. The mean difference between the capillary and venous measurements for CRP was −0.25 mg/dL with 95% limits of agreement of −2.1 mg/dL to 1.6 mg/dL.

**Conclusions:**

POC CRP testing via capillary sampling by finger prick demonstrated sufficient accuracy. POC CRP testing has the potential to be a valuable instrument for clinical decision making, particularly in screening febrile outpatient children.

## Graphical abstract

Bland‐Altman plot for comparison between POC testing using capillary blood samples and standard venous blood tests for CRP levels.

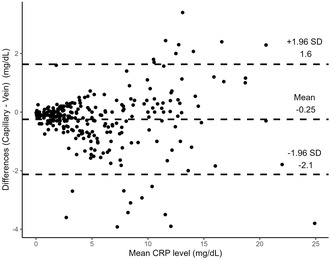

## 1 INTRODUCTION

Pyrexia is a prevalent manifestation among pediatric outpatients. Although the incidence of severe bacterial infections is low, prompt identification is crucial to reduce the risk of life‐threatening complications. White blood cell (WBC) count and C‐reactive protein (CRP) levels rapidly increase in response to infectious agents, inflammatory processes, or neoplastic conditions. Assessing WBC counts and CRP levels in outpatients can help diagnose severe infections in the pediatric population. CRP plays a crucial role in differentiating bacterial from viral etiologies.^1^ CRP has been shown to be a useful marker for differentiating bacterial pneumonia from nonbacterial pneumonia and predicting the severity of pneumonia‐related complications in patients with suspected bacterial pneumonia.^2^, ^3^ However, venipuncture in infants poses greater challenges than that in adults and requires specialized proficiency. Measuring CRP levels using blood collected by venipuncture is less feasible as a routine screening test because of the pain it causes in pediatric patients. Conversely, capillary blood sampling for point‐of‐care (POC) testing is a less painful and easier blood sampling technique than venous blood sampling.

Comparative analyses of CRP levels between POC testing and standard central laboratory testing using identical venous samples have shown concordance.^4^ However, studies on the accuracy of POC testing using capillary samples obtained through finger prick in the pediatric outpatient setting are rare.^5^, ^6^, ^7^ Because the smaller size of infants and their likelihood of uncooperative behavior may influence the test results, establishing the accuracy of POC testing using capillary samples in the pediatric clinical setting is necessary. Studies conducted in the Netherlands and the UK have shown that using POC CRP testing for children is rare compared with adults.^8^ This may be because of concerns about the accuracy of POC CRP testing. Its diagnostic value in children has not been recognized by general practitioners.^9^ Therefore, this study aimed to evaluate the analytical accuracy of POC testing for WBC and CRP using capillary blood samples compared with standard central laboratory tests using venous blood samples in a pediatric primary care setting.

## 2 MATERIALS AND METHODS

### 2.1 Study design

This was a retrospective study conducted on outpatients at the Department of Pediatrics of Tsugaruhoken Medical COOP Kensei Hospital. This was conducted in accordance with the Declaration of Helsinki. This study was approved by the Ethics Committee of Tsugaruhoken Medical COOP Kensei Hospital (approval number: N/A). Given the study's retrospective nature, we used an opt‐out consent process as approved by our ethics committee. Information about the study was publicly posted on our institution's website and in the outpatient waiting area. This disclosure included the study's purpose and methods and emphasized each patient's right to opt out without any impact on his or her medical care. Patients or their legal representatives could opt out by phone, email, or in person. To protect patient privacy, all data were stored on secure, password‐protected servers accessible only to authorized research personnel.

This study included individuals under the age of 18 years with clinical manifestations indicative of infectious or inflammatory diseases who underwent capillary and subsequent venous blood sampling for WBC and CRP testing within a 2‐h window as part of their diagnostic workup between January 2011 and February 2022.

### 2.2 Data collection

Capillary and venous blood samples were collected from each patient within a 2‐h interval by a dedicated pediatric nurse. Capillary blood samples were collected by finger prick. Venous blood samples were collected through the conventional venipuncture technique. POC testing on the capillary blood samples was performed using Microsemi LC‐667CRP “HORIBA” until October 31, 2018, and Microsemi LC‐767CRP “HORIBA” subsequently. The Microsemi CRP system (Horiba Medical, Kyoto, Japan) requires 60 μL of EDTA‐2 K anticoagulated whole blood for the simultaneous measurement of complete blood count with 3‐Diff and CRP within approximately 4 min.^4^ Both samples were measured by certified laboratory technicians in the hospital's central laboratory.

### 2.3 Data analysis

All statistical analyses were performed using the R software (version 4.3.2). Data from both samples, including WBC count, CRP level, hematocrit level, and platelet count, were presented as mean and standard deviation. We used the Bland‐Altman method to determine whether measures made using the two measurement techniques agreed. This approach allowed us to visualize the mean difference and 95% limits of agreement (LoA) between the methods. The 95% LoA was calculated as the mean difference ± 1.96 standard deviations of the differences. The two measurement techniques were considered clinically equivalent if the 95% LoA range was acceptable. Based on a previous study,^1^ positive and negative predictive values of capillary blood sampling in relation to venous blood sampling for cutoff values higher than 8.0 mg/dL and lower than 2.0 mg/dL were calculated.

## 3 RESULTS

**FIGURE 1:**
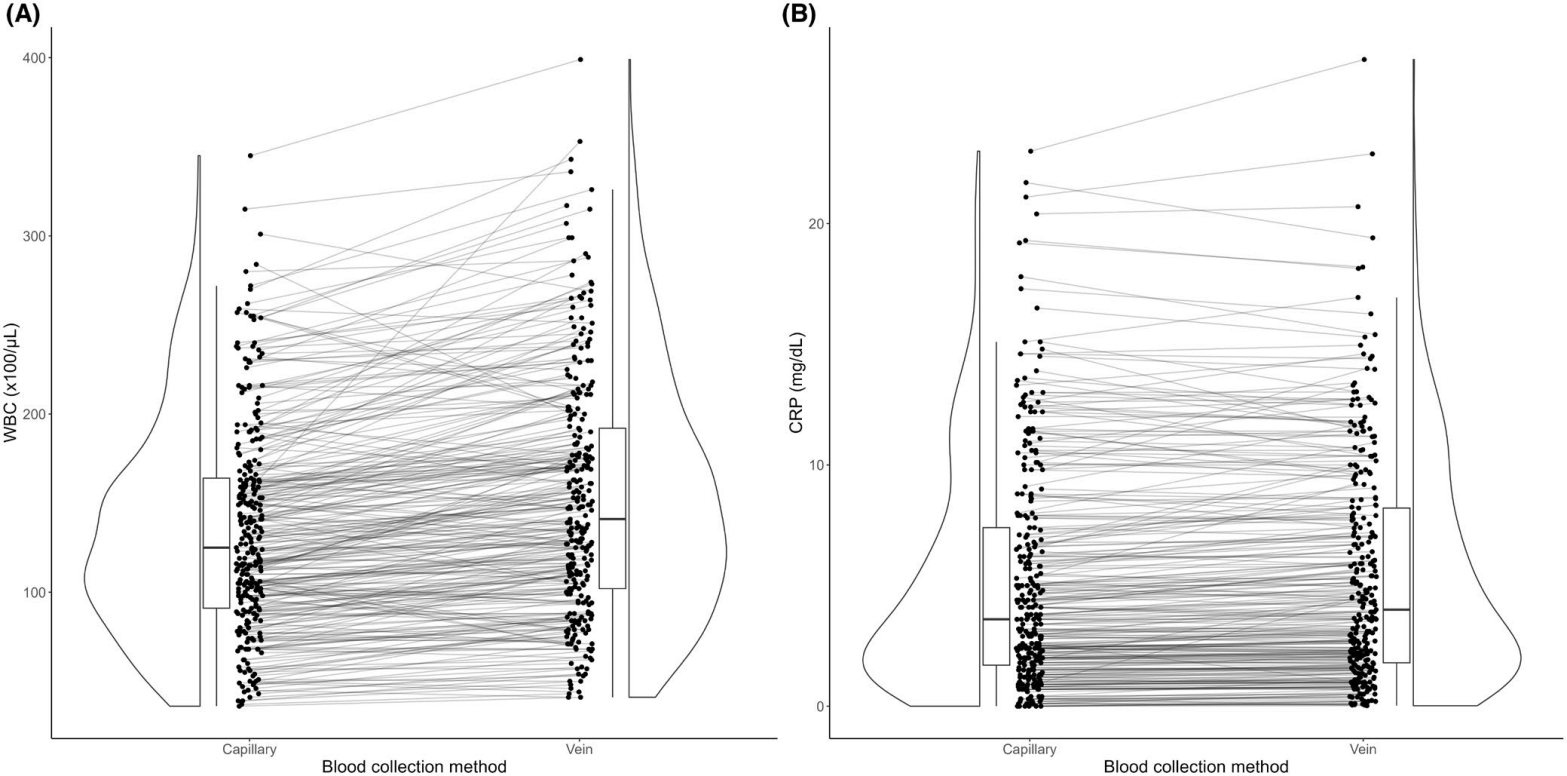
Raincloud plot for comparison between point‐of‐care testing using capillary blood samples and standard venous blood tests. (A) White blood cell (WBC) count. (B) C‐reactive protein (CRP) level.

**FIGURE 2:**
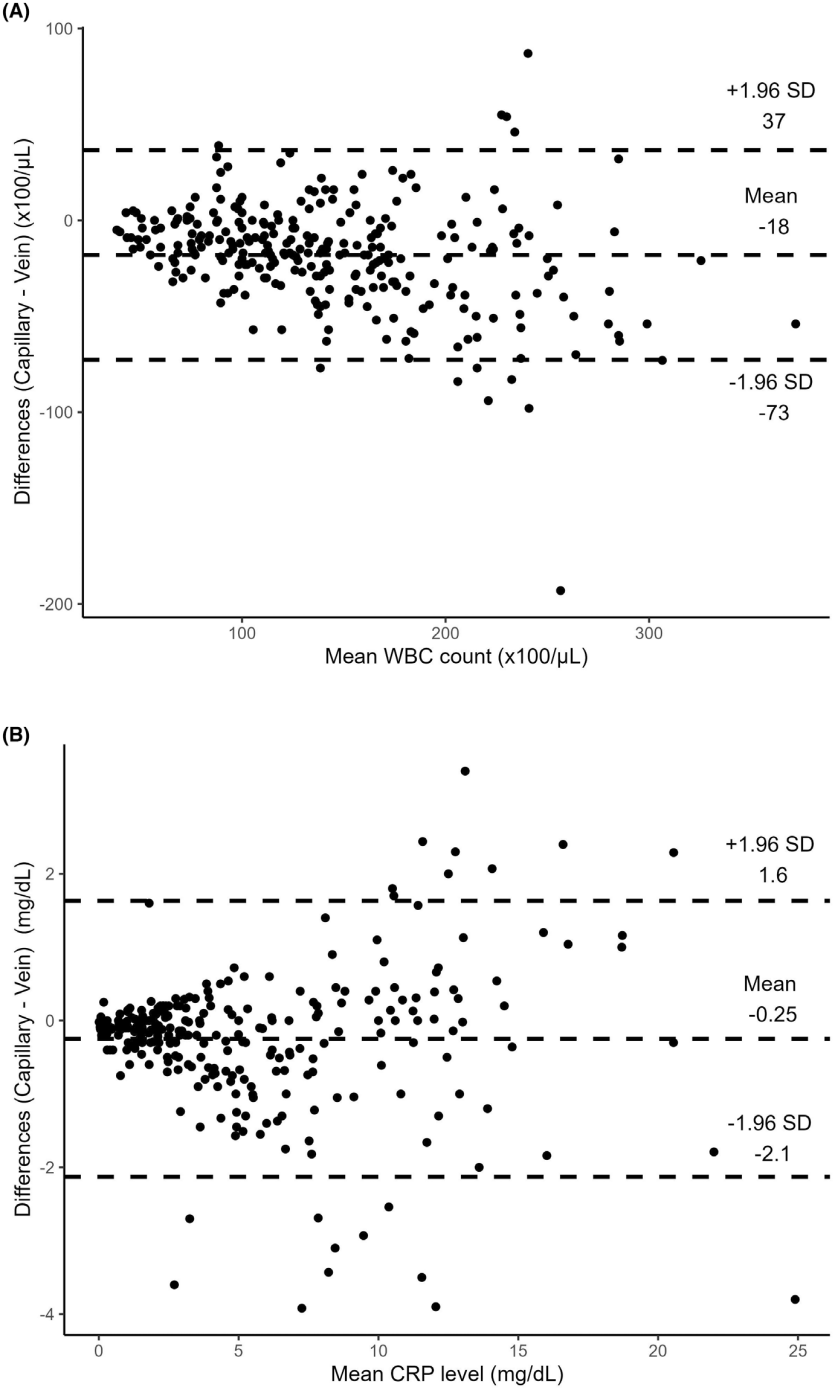
Bland‐Altman plot for comparison between point‐of‐care testing using capillary blood samples and standard venous blood tests. (A) White blood cell (WBC) count. (B) C‐reactive protein (CRP) level. SD, standard deviation.

A total of 277 pediatric patients were included in this study. The median age was 1 year (interquartile range: 0–2 years). Of the 277 patients, 145 were males (52%). Table 1 shows the WBC counts, CRP levels, hemoglobin levels, hematocrit levels, and platelet counts in the capillary and venous blood samples. Platelet counts in capillary blood samples were lower than those in venous blood samples. Figure 1 shows WBC counts and CRP levels in capillary versus venous blood samples. The accuracy of POC testing for WBC and CRP using capillary blood samples compared with venous samples was evaluated using Bland‐Altman analysis. The mean difference between the capillary and venous measurements for WBC was −18 × 100/μL with 95% LoA of −73 × 100/μL to 37 × 100/μL (Figure 2A). The variation in mean difference tended to increase for WBC counts above 15 × 100/μL. The mean difference between the capillary and venous measurements for CRP was −0.25 mg/dL with 95% LoA of −2.1 mg/dL to 1.6 mg/dL (Figure 2B). The variation in mean difference tended to increase for CRP levels above 5.0 mg/dL.

**TABLE 1:** Characteristics of laboratory data included in the study.

	Capillary blood (*n* = 277)	Venous blood (*n* = 277)
WBC, mean (SD)	134.5 (59.5)	152.6 (68.2)
CRP, mean (SD)	5.2 (4.7)	5.5 (4.7)
Hemoglobin, mean (SD)	12.0 (1.4)	11.7 (1.1)
Hematocrit, mean (SD)	35.9 (3.9)	35.1 (3.0)
Platelets, mean (SD)	21.6 (9.9)	33.9 (12.8)

Abbreviation: SD, standard deviation.

The positive and negative predictive values of capillary blood samples in relation to venous blood sampling for cutoff values of 8.0 mg/dL or higher were 97% (62/64) and 96% (204/213), respectively. The sensitivity and specificity of the capillary CRP are 87% (62/71) and 99% (204/206). The positive and negative predictive values of capillary blood sampling in relation to venous blood sampling for cutoff values of 2.0 mg/dL or lower were 87% (75/86) and 99% (190/191), respectively. The sensitivity and specificity of the capillary CRP are 99% (75/76) and 95% (190/201).

## 4 DISCUSSION

This study demonstrates that the POC CRP test results obtained from finger‐prick capillary sampling are comparable to conventional central laboratory test results obtained from venous blood sampling. This is because the 95% LOA range (−2.1 mg/dL to 1.6 mg/dL) was considered narrow enough to be clinically relevant. This finding indicates that POC CRP testing is a reliable modality for clinical decision making in pediatric primary care. Conversely, the WBC counts obtained from the capillary samples did not exhibit equivalent reliability, as indicated by the wide range of 95% LoA (−73 × 100/μL to 37 × 100/μL). However, the WBC counts obtained from the capillary samples may, however, be relatively reliable up to a WBC count of 10 × 100/μL, as the Bland‐Altman plots show that the mean differences between the two measurement techniques appear small.

POC CRP testing using capillary sampling is considered a sufficiently useful screening tool. Previous studies have shown that measurement results obtained using capillary sampling in the clinical setting are comparable to test results obtained with venous blood sampling, with high agreement and accuracy.^5^, ^6^, ^7^ In practice, when the POC CRP level is high, additional tests, such as venous blood analysis, blood cultures, and urinalysis, are often performed. Therefore, small errors are probably inconsequential within the clinical context.

To the best of our knowledge, this is the first study to evaluate the analytical accuracy of CRP from capillary samples using Microsemi CRP with venous sampling as the referential standard. The Microsemi CRP measurement method is based on the immunoturbidimetric assay. Previous studies have shown substantial concordance between Microsemi CRP and conventional tests employing identical venous samples.^4^ A previous study comparing eight commercially available methods reported that the analytical accuracy of Microsemi was so reliable that it was comparable to other analyzers.^10^ This study showed that Microsemi CRP from capillary samples with finger prick yielded accurate results in a pediatric ambulatory care setting.

Conversely, POC WBC testing was considered unreliable, with discrepancies between the results from capillary and venipuncture sampling. This finding is consistent with that of previous studies that showed that the POC WBC count with capillary sampling was lower than that with standard testing with venous sampling.^5^ According to that study, the WBC counts of the POC and laboratory tests were in good agreement when the same venous samples were tested. However, one study on healthy adults showed no clinically relevant differences between venous and finger puncture blood specimens.^11^ Thus, we inferred that POC WBC testing could be more affected by the sampling technique of pricking the finger than POC CRP testing.

Differences in blood collection methods between capillary sampling with finger prick and venipuncture sampling may affect the results. Capillary sampling has technical problems, such as squeezing the finger too hard.^5^ In this study, most of the patients were infants, and sample collection in a crying and resisting situation may have affected the results. This study showed that the blood collection method may have had a greater impact on POC WBC testing than on POC CRP testing.

POC testing using capillary samples has several advantages. First, capillary sampling by finger prick is minimally invasive. Second, the finger‐prick technique is less complex and more readily mastered than venipuncture, with a reduced incidence of collection failures. Third, the duration required for capillary blood sampling is notably less than that for venous sampling. The test results are available faster than those of conventional laboratory testing using venous samples. Consequently, the patient's outpatient stay is shorter.

This study has several limitations. First, temporal disparities in the acquisition of blood samples may have influenced the heterogeneity of the results because the timing of capillary and venous blood sample collection was not precisely concurrent. Second, the study participants were restricted to patients who required subsequent venous blood sampling following initial capillary blood extraction. Thus, there may be selection bias. Third, we recommend cautiously interpreting our positive and negative predictive values, as they are heavily influenced by the condition's prevalence within the target population. In our study population, the mean CRP level was >5.0 mg/L, and approximately 25% of participants had CRP levels that exceeded 8.0 mg/L. This distribution may not accurately reflect the true prevalence in the general population. Nevertheless, given the high sensitivity and specificity of both cutoff values (2.0 mg/L and 8.0 mg/L), their combined use may provide a reasonable foundation for outpatient clinical decision making. Fourth, we did not establish the acceptable LoA for the Bland‐Altman analysis a priori. This decision was made because of the lack of established standards for comparing capillary and venous WBC counts, and the positioning of POC testing as a screening tool rather than a replacement for central laboratory testing. Instead, we focused on identifying trends in the differences between the two testing methods. Future studies may benefit from establishing such benchmarks based on clinical relevance and the specific context of use. Fifth, while the stress of capillary blood sampling could theoretically affect subsequent venous blood sampling results, we believe this effect to be minimal because of the quick nature of the capillary sampling procedure.

In conclusion, POC CRP testing using capillary samples obtained by finger prick demonstrated adequate accuracy. POC CRP testing may be a valuable tool in clinical decision making, particularly as a screening modality for febrile pediatric outpatients.

## FUNDING INFORMATION

The authors declare no funding.

## CONFLICT OF INTEREST STATEMENT

The authors declare no conflicts of interest.

## ETHICS APPROVAL STATEMENT

This study was conducted in accordance with the Declaration of Helsinki. This study was approved by the Ethics Committee of Tsugaruhoken Medical COOP Kensei Hospital.

## Data Availability

Data are not shared.
